# Resveratrol Mediated Modulation of Sirt-1/Runx2 Promotes Osteogenic Differentiation of Mesenchymal Stem Cells: *Potential Role of Runx2 Deacetylation*

**DOI:** 10.1371/journal.pone.0035712

**Published:** 2012-04-23

**Authors:** Mehdi Shakibaei, Parviz Shayan, Franziska Busch, Constance Aldinger, Constanze Buhrmann, Cora Lueders, Ali Mobasheri

**Affiliations:** 1 Institute of Anatomy, Ludwig-Maximilian-University Munich, Munich, Germany; 2 Investigating Institute Molecular Biological System Transfer, Tehran, Iran; 3 Department of Thoracic and Cardiovascular Surgery, Laboratory for Tissue Engineering, German Heart Institute Berlin, Berlin, Germany; 4 Division of Veterinary Medicine, School of Veterinary Medicine and Science, Faculty of Medicine and Health Sciences, University of Nottingham, Sutton Bonington Campus, Sutton Bonington, United Kingdom; Institut de Génomique Fonctionnelle de Lyon, France

## Abstract

**Objective:**

Osteogenic repair in response to bone injury is characterized by activation and differentiation of mesenchymal stem cells (MSCs) to osteoblasts. This study determined whether activation of Sirt-1 (a NAD^+^-dependent histone deacetylase) by the phytoestrogen resveratrol affects osteogenic differentiation.

**Methods:**

Monolayer and high-density cultures of MSCs and pre-osteoblastic cells were treated with an osteogenic induction medium with/without the Sirt-1 inhibitor nicotinamide or/and resveratrol in a concentration dependent manner.

**Results:**

MSCs and pre-osteoblastic cells differentiated to osteoblasts when exposed to osteogenic-induction medium. The osteogenic response was blocked by nicotinamide, resulting in adipogenic differentiation and expression of the adipose transcription regulator PPAR-γ (peroxisome proliferator-activated receptor). However, in nicotinamide-treated cultures, pre-treatment with resveratrol significantly enhanced osteogenesis by increasing expression of Runx2 (bone specific transcription factor) and decreasing expression of PPAR-γ. Activation of Sirt-1 by resveratrol in MSCs increased its binding to PPAR-γ and repressed PPAR-γ activity by involving its cofactor NCoR (nuclear receptor co-repressor). The modulatory effects of resveratrol on nicotinamide-induced expression of PPAR-γ and its cofactor NCoR were found to be mediated, at least in part, by Sirt-1/Runx2 association and deacetylation of Runx2.

Finally, knockdown of Sirt-1 by using antisense oligonucleotides downregulated the expression of Sirt-1 protein and abolished the inhibitory effects of resveratrol, namely nicotinamide-induced Sirt-1 suppression and Runx2 acetylation, suggesting that the acetylated content of Runx2 is related to downregulated Sirt-1 expression.

**Conclusion:**

These data support a critical role for Runx2 acetylation/deacetylation during osteogenic differentiation in MSCs *in vitro*. (*242 words in abstract*)

## Introduction

Mesenchymal stem cells (MSCs) are multipotent cells that can differentiate into distinct connective tissue cell types (i.e. osteoblasts, chondroblasts, adipocytes, myoblasts, etc.) [1], [2]. MSCs may be used in tissue engineering to restore or replace tissues and organs. Although bone marrow is a good source for MSCs, the cells are available in limited quantities [2]. An alternative source for MSCs is adipose tissue; adipose derived MSCs can differentiate down the adipogenic, chondrogenic, myogenic, neurogenic, and osteogenic cell lineage pathways [3]. However, more detailed information about differentiation of MSCs to osteoblasts *in vitro* is essential for the understanding and treatment of bone regeneration and osteoporosis. In age-related osteoporosis, adipocytes are increased in bone marrow [4]. It is known that osteoporosis is linked with estrogen deficiency after menopause and this is one of the most common causes of age-related bone loss [5]. Hormone replacement therapy (HRT) inhibits endocrine-deficient postmenopausal osteoporosis and can reduce the incidence of bone fractures [6], but adverse side effects of these drugs have recently come to light. HRT increases the risk of developing breast and endometrial cancer [7] and has other undesirable side effects including fluid retention, headaches, mood swings and depression, which can significantly reduce quality of life in women. Therefore, safer, natural and more selective pharmacotherapies and natural remedies for menopause-induced osteoporosis are needed.

Resveratrol is a polyphenolic phytoestrogen (trans-3,5, 4′-trihydroxystilbene) found in the skin of red grapes, red vines, various other fruits, peanuts and root extracts of *Polygonum cuspidatum* [8]. Resveratrol acts as a mixed agonist/antagonist for the estrogen receptors alpha and beta [9]. Through binding to the estrogen receptor, resveratrol is thought to exert beneficial effects on the cardiovascular system and may reverse osteoporosis by a direct stimulatory effect on bone formation in osteoblastic cells [10]. Many of the biological effects of resveratrol have already been demonstrated in the literature; these include cardiovascular protection [11], anticancer activity [12] and stimulation of proliferation and osteoblastic differentiation in human and mouse MSCs [13], [14]. However, its effects on bone are less studied and are particularly relevant to this investigation.

The sirtuins (silent information regulator 2- Sir2) are highly conserved nicotinamide adenine dinucleotide (NAD)-dependent enzymes that deacetylate residues of acetylated lysine. These histone deacetylases (HDAC) are involved in deacetylation of histones and non-histone proteins, including transcription factors, proteins and enzymes playing an important role in chromatin architectures, gene expression, control of cellular metabolism and cancer in many species [15], [16]. Mammals possess seven sirtuins (SIRTs), whereas the histone deacetylase Sirt-1 is located in the nucleus and shares identity with Sir2 [17]. The activity of the Sirt-1 protein is known to be regulated by resveratrol and nicotinamide, which activate and inhibit Sirt-1, respectively [18]. Activation of Sirt-1 decreases adipocyte formation during osteoblastic differentiation of MSCs [14], [19].

PPAR-γ, a member of the nuclear receptors has been found to be an important regulator of adipogenesis and plays a central role in fat tissue development [20], inflammatory responses, cellular proliferation and differentiation [21], as well as the balance between osteogenesis and adipogenesis [22]. PPAR-γ is activated by a wide variety of substances including long chain fatty acids, peroxisome proliferators and thiazolidinedione compounds [23]. In addition, it has been shown that adipocytes and osteoblasts share a common progenitor, i.e., mesenchymal stem cells in which expression of PPAR-γ signaling can induce transdifferentiation of osteoblasts to adipocytes in adipogenic medium [24]. Moreover, several nuclear receptors have been found to interact with the nuclear receptor co-repressor (NCoR) and silencing mediator for retinoid and thyroid hormone receptor (SMRT) [25]. Furthermore, these co-repressors are required for the inhibition function of nuclear receptors and transcription factors [26].

The transcription factor, Runt-related transcription factor 2 (Runx2) is one of the earliest and most specific markers during osteogenesis. Runx2 induces osteoblast-specific gene expression *in vitro* [14], [27]. Different specific signals, like mechanical signals can regulate Runx2 activation stimulating osteoblast differentiation through the activation of the MAPKinase signal-transduction pathway and Ras/Raf-dependent Erk1/2 activation [28].

In this study, we have established an *in vitro* model of osteogenesis using adipose derived MSCs in monolayer and high-density cultures and present new evidence to show that resveratrol-activated Sirt-1 significantly favors osteogenic differentiation over adipogenic differentiation. The question of whether an interaction between resveratrol-activated Sirt-1 and Runx2 occurs and whether this causes deacetylation of Runx2 during osteogenesis is an important focus of this study.

## Materials and Methods

### Antibodies

Polyclonal anti-collagen type I antibody and alkaline phosphatase linked sheep anti-mouse and sheep anti-rabbit secondary antibodies for immunoblotting were purchased from Millipore (Schwalbach, Germany). Polyclonal anti- Runx2 was purchased from Alpha Diagnostics Int. San Antonio, TX, USA. Monoclonal anti-β-actin and nicotinamide were purchased from Sigma-Aldrich (Munich, Germany). Polyclonal anti-Sirt-1 and anti-NCoR were purchased from Abcam PLC (Cambridge, UK). Polyclonal anti-PPAR-γ antibodies were purchased from Acris Antibodies GmbH, Germany. Acetylated-lysine (Ac-K-103) antibody was purchased from Cell Signaling Technology (Danvers, MA, USA).

Resveratrol with purity greater than 98% was purchased from Sigma-Aldrich (Munich, Germany). A 100-mM stock solution of resveratrol (molecular weight, 228.2) was prepared in ethanol and further diluted in cell culture medium to prepare working concentrations. The maximum final content of ethanol in cultures was less than 0.1%. This concentration was also used as a control.

### Isolation and culture of mesenchymal stem cells

Mesenchymal stem cells were isolated from canine adipose tissue biopsies obtained during orthopedic surgeries (from 3 animals between the age of ca. 5–7 years), as previously described [29]. Fully informed owner consent was obtained and the project was approved by the Ludwig-Maximilian University Ethical Review committee. Briefly, adipose tissue was cut into small pieces and digested with collagenase 0.2% in Ham's-F12 in a water bath at 37°C for 2 hours. Digested adipose tissue was centrifuged at 1000 g/5 min and the pellet was resuspended in cell culture medium consisting of DMEM/Ham's-F12 1∶1, 10% FCS, 1% partricin solution, 1% penicillin/streptomycin solution (10 000 IU/10 000 IU), 75 µg/ml ascorbic acid, 1% essential amino acids and 1% Glutamine, all obtained from Seromed (Munich, FRG). The cells were seeded in a T75 cell culture flask and incubated at 37°C/5%CO_2_, 95% humidity. After four days, non-adherent cells were discarded by washing with Hank's salt solution. The medium was changed three times per week. Adherent cells were split following formation of fibroblast-like cell colonies and upon reaching 60–70% confluence, and were sub-cultured until the third or fourth passage was achieved.

#### Pre-osteoblastic cell line culture

The mouse pre-osteoblastic cell line MC3T3-E1 (DSMZ, Braunschweig, Germany) was selected as an *in vitro* model of pre-osteoblastic cells, as previously described [30]. The cells were cultured in alpha-MEM containing 10% FCS, 100 U/mL penicillin and 100 mg/mL streptomycin. The cells were maintained in a humidified, 95% air/5% CO2 atmosphere at 37°C. All experiments were performed with third passage MC3T3-E1 cells. For induction of the osteoblast phenotype, cells were cultured in differentiation medium (DMEM containing 10% FCS, 10 mm β-glycerophosphate and 50 mg/mL ascorbate-2-phosphate) [31].

### Experimental design

Osteogenic differentiation was performed in monolayer culture or in high-density mass culture. Mesenchymal stem cell cultures and pre-osteoblastic MC3T3-E1 cells were either left untreated, or incubated with one of the following treatments: 0.1, 1 and 10 µM resveratrol only; 1, 10 and 100 mM nicotinamide only; pre-stimulated with resveratrol 1 µM for 4 h in suspension and then brought into monolayer or high-density cultures and stimulated with 1, 10 and 100 mM nicotinamide for the indicated time periods. For monolayer culture 10,000 cells were seeded per well in a four-well-plate and cultured until they reached confluency. Cultures were treated as described below in osteogenic induction medium and evaluated after 21 days. The high-density mass culture was performed using procedures and specialized equipment as previously described [32]. Briefly, an 8 µl drop of cells was placed on a cellulose filter on top of a steel mesh bridge, containing about 1 million cells. The osteogenic induction medium was prepared as described by [2], consisting of DMEM base medium, 10% FCS, penicillin/streptomycin solution (10000 IU/10000 IU/100 ml), 10^−7^ M dexamethasone (Sigma-Aldrich, Cat. No. D-8893), 10 mM β-glycerophosphate (Sigma-Aldrich, Cat. No. G-9891) and 50 µM ascorbate-2-phosphate (Sigma-Aldrich, Cat. No. A-8960). Medium changes were made every three days. For the negative control, cells were cultured in cell culture medium containing 10% FCS. To osteogenic induction medium, 0.1, 1 and 10 µM resveratrol and/or 1, 10 and 100 mM nicotinamide were added for the indicated time periods. Cells were nurtured through diffusion at the filter medium interface and evaluated after indicated time periods.

### Light microscopy

Monolayer cultures were stained with von Kossa for mineralized matrix deposition or stained with Oil Red O solution to visualize the formation of fat vacuoles as previously described [33].

### Antisense and lipofectin-mediated transfection

The Sirt-1 antisense sequences used in these experiments were designed using a computational neural network mode [34]. MSCs were plated in 3 cm^2^ tissue culture dishes or in a four-well glass plate at a concentration of 3×10^5^ cells/dish or 1×10^4^ cells/well and were grown to confluence. All transfection experiments were carried out on 50% confluent monolayer cultures. Antisense oligonucleotide sequence (5′-GTATTCCACATGAAACAGACA-3′) was derived from the nucleotide at position 844 to 864 lying in upstream region of the nucleotide sequences coding for the catalytic domain of Sirt-1 mRNA registered under accession number NM012238 in GenBank. To overcome the rapid degradation of antisense sequence by intracellular endo- and exonucleases, the non-bridging oxygen on the phosphate linkage was replaced with a sulfur atom (phosphothioate modification). The phosphothioate modified sense oligonucleotide sequence (5′-TGTCTGTTTCATGTGGAATAC-3′), complementary to the antisense sequence, was used as control. The modified oligonucleotides were purchased from MWG (Ebersberg, Germany). To provide enhanced transfection of oligonucleotides to the cytoplasm of the target cells, lipofectin reagent (Life Technologies, Invitrogen, Darmstadt, Germany) was used according to the manufacturer's instructions. Briefly, 10 µl lipofectin was mixed with 1, 0.5 and 0.2 µM of sense or antisense oligonucleotide (1000 µM) respectively for 30 min at AT and subsequently the mixture was added to 990 µl serum-free medium to obtain a working medium with 1, 0.5 and 0.2 µM of the corresponding oligonucleotide. The medium was then added to the already prepared cells (50–60% confluent) and incubated for 24 h at 37°C. After 24 h of incubation (colonies were pooled from each transfection condition and used in the subsequent experiments), transfection media was replaced by the regular culture or osteogenic induction media and evaluated after 21 days.

### Electron microscopy

Transmission electron microscopy was performed as previously described [35]. Briefly, high-density cultures were fixed for one hour in Karnovsky's fixative and then post-fixed in 1% O_s_O_4_ solution. After dehydration, pellets were embedded in Epon, ultrathin cuts made on a Reichert-Ultracut E. and contrasted with a mixture of 2% uranyl acetate/lead citrate. A transmission electron microscope (Zeiss TEM900, Jena, Germany) was used to examine the cultures.

To quantify adipocyte formation, the number of cells exhibiting typical morphological features like multiple fat vacuoles was determined by scoring 100 cells from 20 different microscopic fields per culture and the number of adipocytes was expressed as an indicator of adipogenic differentiation of MSCs.

### Immunofluorescence analysis of Sirt-1

The effect of specific Sirt-1 antisense or sense on the Sirt-1 expression was investigated by an immunofluorescence method as previously described in detail [36]. Briefly, the MSCs were cultured in 4-well glass plates and incubated for 24 h. Serum-starved cells were treated with 1 µM end concentration of antisense or sense for 24 hours in serum-starved medium. Glass plates were rinsed three-times in Hanks solution before methanol fixation for 10 min at ambient temperature (AT), and rinsing with PBS. Cell membranes were permeabilized by treatment with 0.1% Triton X-100 for 1 min on ice. Cells were overlaid with protease-free bovine serum albumin (BSA) for 10 min at AT, rinsed with PBS and incubated with primary antibodies (Sirt-1, 1∶30 in PBS) in a humid chamber overnight at 4°C. They were gently washed several times with PBS before incubation with rhodamine-red conjugated secondary antibody for 2 h at AT and finally washed again three times with Aqua Dest laboratory water. Counterstaining was performed with DAPI to visualize the cell nuclei. Samples were evaluated under light microscope (Leica, Germany) and photomicrographs were digitally captured and stored.

### Immunoprecipitation and Immunoblotting

A detailed description of the technique used for the following experiments has been previously published [37], [38]. Briefly, high-density cultures were rinsed in PBS and the proteins extracted with lysis buffer (50 mM Tris/HCl (pH 7.2), 150 mM NaCl, l% (v/v) Triton X-100, 1 mM sodium orthovanadate, 50 mM sodium pyrophosphate, 100 mM sodium fluoride, 0.01% (v/v) aprotinin, pepstatin A (4 µg/ml), leupeptin (10 µg/ml) and 1 mM phenylmethylsulfonyl fluoride (PMSF)) for 30 min on ice. After adjusting the total protein concentration, samples were separated by SDS-PAGE (5%–12% gels) under reducing conditions. For immunoprecipitation, the extracts were pre-cleared by incubating them first with 25 µl of either normal rabbit IgG-serum or normal mouse IgG-serum and *Staphylococcus (S.) aureus* cells, then with primary antibodies diluted in wash buffer (0.1% Tween 20, 150 mM NaCl, 50 mM Tris-HCl (pH 7.2), 1 mM CaCl_2_, 1 mM MgCl_2_ and 1 mM PMSF) for 2 h at 4°C, and finally with *S. aureus* cells for 1 h at 4°C. Control immunoprecipitations were performed by incubating the samples with non-immune rabbit anti-mouse IgG alone. *S. aureus* cells were washed five times with wash buffer and once with 50 mM Tris-HCl (pH 7.2) and then boiled in SDS-PAGE sample buffer. Separated proteins were transferred to nitrocellulose membranes and incubated in blocking buffer (5% (w/v) skimmed milk powder in PBS/0.1% Tween 20) for 1 h at AT. Membranes were incubated overnight with the first antibody diluted in blocking buffer at 4°C on a shaker, washed three times with blocking buffer, and then incubated with the secondary antibody conjugated with alkaline phosphatase for 90 min at AT. Membranes were rinsed with blocking buffer and then washed three times in 0.1 M Tris (pH 9.5) containing 0.05 M MgCl_2_ and 0.1 M NaCl. Specific antigen-antibody complexes were rendered visible using nitro-blue tetrazolium and 5-bromo-4-chloro-3-indoylphosphate (p-toluidine salt; Pierce, Rockford, IL, USA) as the substrates for alkaline phosphatase. Total protein concentration was determined according to the bicinchoninic acid system (Pierce, Rockford, IL, USA) using bovine serum albumin as a standard. Specific binding was quantified by densitometry using “quantity one” (Bio-Rad Laboratories Inc. CA, USA).

### Runx2 acetylation assay

Runx2 lysine acetylation was analyzed by immunoprecipitation of Runx2 followed by western blotting using acetyl-lysine antibodies. Cells were treated with 1 µM resveratrol for 4 hours and then exposed to 1, 10 and 100 mM nicotinamide for indicated times. Whole-cell extracts were prepared, immunoprecipitated with an anti- Runx2 antibody, and subjected to western blot analysis using an anti–acetyl-lysine antibody. To confirm these observations, MSCs were treated with 1 µM resveratrol for 4 h and then transfected with specific Sirt-1 antisense or sense oligonucleotides at 1 µM end concentration for 24 h. After 24 h of incubation transfection media were replaced with the regular culture medium or osteogenic induction medium with or without nicotinamide (10 mM) and evaluated after 21 days. Whole cell extracts were prepared and subjected to immunoprecipitation with anti- Runx2 antibody and the precipitates were separated by SDS-PAGE and immunoblotted using antibodies against acetyl-lysine and Runx2.

### Co-Immunoprecipitation of Runx2 and Sirt-1

Endogenous protein interactions from high-density cultures were evaluated by co-immunoprecipitation experiments using Sirt-1 and Runx2 antibodies. Cells were treated with 1 µM resveratrol for 4 hours and then exposed to 1, 10 and 100 mM nicotinamide for the indicated times. Whole-cell extracts were prepared, immunoprecipitated with an anti- Runx2 antibody, and precipitates were subjected to western blot analysis using an anti–Sirt-1 antibody. To confirm the protein-protein interactions in MSCs, cells were treated with 1 µM resveratrol for 4 hours and then transfected with 1 µM end concentration of specific Sirt-1 antisense or sense oligonucleotides for 24 h. After 24 h of incubation transfection media were replaced by the regular culture medium or osteogenic induction medium with or without nicotinamide (10 mM) and evaluated after 21 days. Whole cell extracts were prepared and subjected to immunoprecipitation with anti-Runx2 antibody and the precipitates were separated by SDS-PAGE and immunoblotted using antibodies against Sirt-1.

### Statistical analysis

Numerical data are expressed as mean values (+/−SD) for a representative experiment performed in triplicate. The means were compared using student's *t*-test assuming equal variances. Differences were considered to be statistically significant if the *P*-value was less than 0.05.

## Results

### Effects of resveratrol or/and nicotinamide on osteogenic differentiation of MSC in monolayer cultures

**Figure 1:**
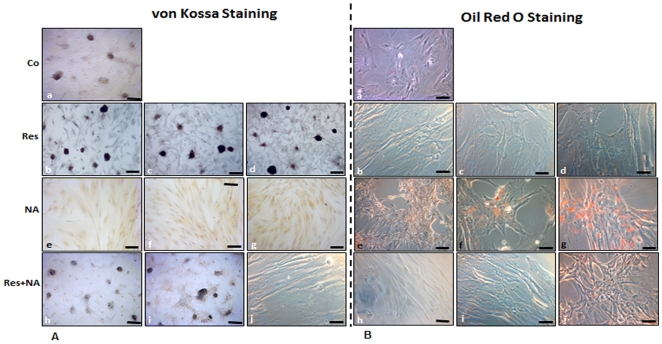
Light microscopic evaluation of the effects of resveratrol and/or nicotinamide on osteoblastic differentiation of MSCs in monolayer culture. *a–d: Light microscopic demonstration of osteoid-tissue formation with von Kossa staining (A) or adipose-tissue formation with Oil Red O staining (B).* 21 days in monolayer culture. In cultures were stimulated with osteogenic induction medium (A,B: a) and with various concentrations of resveratrol (0.1 µM (A,B: b), 1 µM (A,B: c), 10 µM (A,B: d)calcium deposition was observed (A: a–d), but adipogenesis was negative (B: a–d). Light microscopy demonstrated that MSC cultures treated with osteogenic medium and with the sirtuin inhibitor nicotinamide (1 mM (A, B: e), 10 mM (A,B: f) and 100 mM (A,B: g)), did not differentiate to osteoblastic cells (A, e–g), but differentiated into adipocytes (B, e–g), exhibiting cytoplasmic lipid droplet accumulation in the presence of osteogenic induction medium. In another approach, MSCs were pre-treated with 1 µM resveratrol and then co-treated with various concentrations of nicotinamide (1 mM (A,B: h), 10 mM (A,B: i) and 100 mM (A,B: j)) in osteogenic medium. Pre-treatment of MSCs with 1 µM resveratrol and co-treatment with 1 and 10 mM nicotinamide inhibited adipogenic differentiation of MSCs (B: h–i), favoring osteoblastic differentiation (A: h–i). However, co-treatment with 100 mM nicotinamide resulted in adipogenesis (B: j), but not in osteogenesis (A: j). Magnification: ×200, *bar* 30 µm.

Incubation of MSCs in monolayer cultures with osteogenic induction medium over 3 weeks resulted in osteogenesis; positive von Kossa staining and high quantities of calcium deposition (Fig. 1A, a) and negative oil Red O staining (Fig. 1B, a) was observed in MSC cultures. In untreated pure MSC cultures, no calcium deposition was observed (data not shown). Treatment of MSC cultures with the osteogenic induction medium and resveratrol (various concentrations, 0.1, 1 and 10 µM) induced osteogenesis and produced positive von Kossa staining (Fig. 1A, b–d) and negative oil Red O staining (Fig. 1B, b–d). In contrast, in the presence of the sirtuin inhibitor nicotinamide, osteogenesis was not observed (Fig. 1A, e–g). Cells differentiated to adipocytes and contained more vacuoles compared to the resveratrol treated cells. Oil Red O staining for fat deposition revealed the presence of fat vacuoles containing neutral lipids (Fig. 1B, e–g). The number of differentiated adipocytes in culture increased in the presence of 10 or 100 mM nicotinamide. To test whether activation of Sirt-1 inhibits adipogenesis during osteoblastic differentiation [19], [39], MSC cultures were treated with resveratrol and then co-treated with various concentrations of nicotinamide in osteogenic induction medium. Pre-treatment of MSCs with resveratrol and co-treatment with nicotinamide promoted osteogenic differentiation (Fig. 1A, h–i) and inhibited adipogenic differentiation (Fig. 1B, h–i). However, the inhibition of adipogenesis by resveratrol was concentration dependent. Pre-treatment of MSCs with 1 µM resveratrol and co-treatment with 100 mM nicotinamide did not result in osteogenesis (Fig. 1A, j), but stimulated adipogenesis (Fig. 1B, j).

### Effects of resveratrol or/and nicotinamide on osteogenic differentiation of MSC and pre-osteoblastic cells in high-density cultures

**Figure 2:**
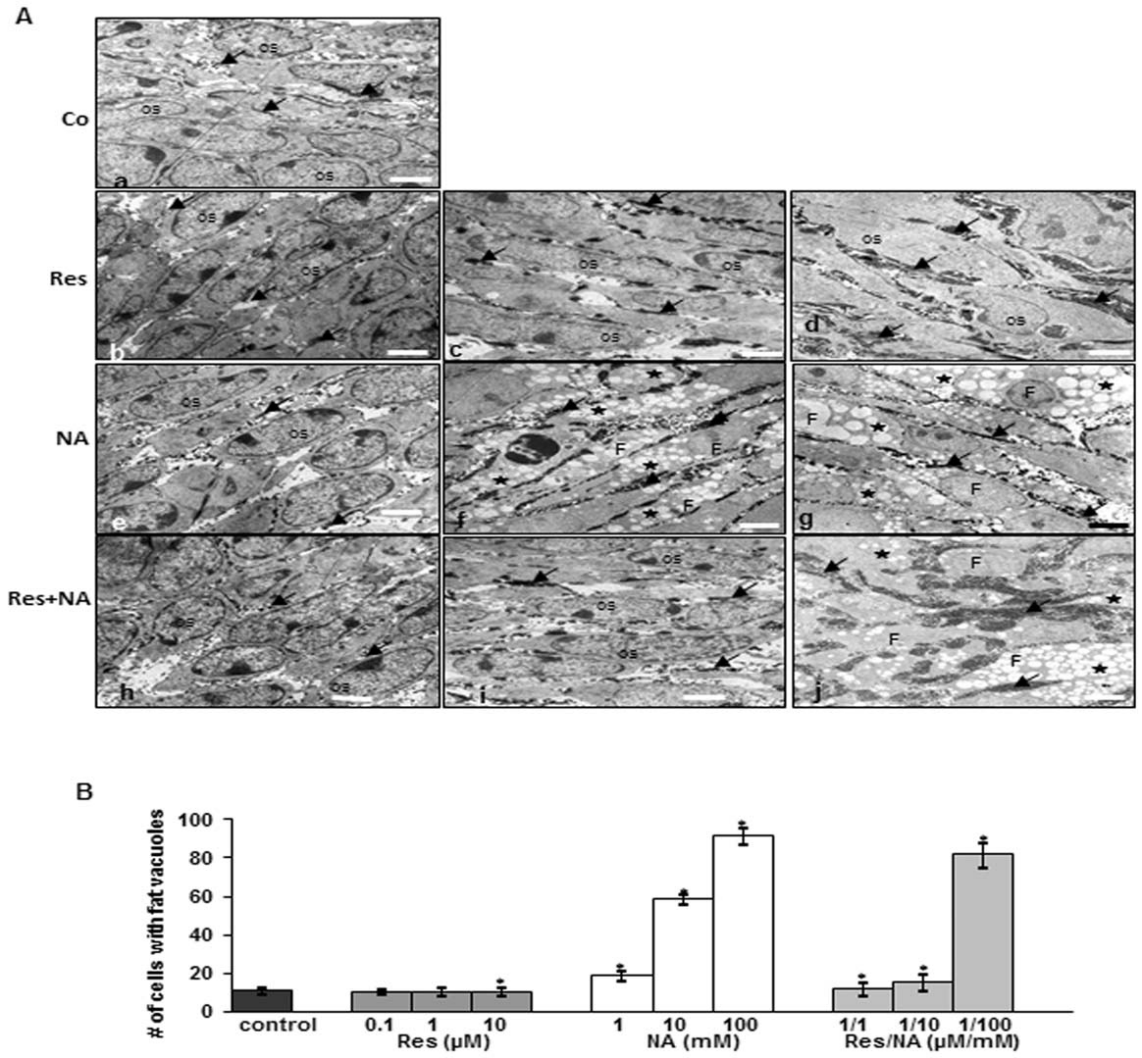
Transmission electron microscopic (TEM) studies of the effects of resveratrol on osteoblastic differentiation of MSCs in high-density culture. *A: a–d: 14 days in high-density culture.* Cultures were stimulated with osteogenic induction medium (a) and with various concentrations of resveratrol (0.1 µM (b), 1 µM (c), 10 µM (d)). TEM revealed details of ultrastructural changes that the MSCs underwent while differentiating into osteoblasts (Os). Cells contained high levels of nuclear euchromatin and a large number of sub-cellular organelles (mitochondria, rough ER, Golgi apparatus). Large quantities of thick extracellular matrix fibrils (arrows) were observed in the extracellular space (arrows). However, no significant differences in osteogenesis were observed at the ultrastructural level between resveratrol-treated and control MSC cultures. Magnification: 5000×, bar = 1 µm. *A: e–g: 14 days in high-density culture.* MSC cultures were treated with osteogenic medium and with the sirtuin inhibitor nicotinamide (1 mM (e), 10 mM (f) and 100 mM (g)). TEM clearly demonstrated that MSCs differentiated into adipocytes (F), exhibiting cytoplasmic lipid droplet accumulation (*) in the presence of osteogenic induction medium. The adipocytes produced high quantities of ECM (arrows) and were embedded in this well organized matrix. Magnification: 5000×, bar = 1 µm. *A: h–j: 14 days high-density culture.* MSCs were pre-treated with 1 µM resveratrol for 4 h and then co-treated with various concentrations of nicotinamide (1 mM (h), 10 mM (i) and 100 mM (j)) in osteogenic medium. Pre-treatment of MSCs with 1 µM resveratrol and co-treatment with 1 and 10 mM nicotinamide inhibited adipogenic differentiation of MSCs, favoring osteoblastic differentiation. However, co-treatment with 100 mM nicotinamide resulted in adipogenesis. Magnification: 5000×, bar = 1 µm. B: Adipocyte differentiation in the cultures was estimated by counting 100 cells from 20 different microscopic fields. The number of adipocytes was highest in cultures stimulated with 100 mM nicotinamide alone. However, cells pre-treated with resveratrol and co-treated with nicotinamide at 1 or 10 mM but not at 100 mM nicotinamide significantly decreased the number of adipocytes compared to the chemical by itself (*).

Incubation of MSCs with osteogenic induction medium resulted in osteogenesis; cells exhibited high levels of nuclear euchromatin, large numbers of morphologically normal cellular organelles (mitochondria, rough ER, Golgi apparatus), numerous cell-cell processes and large quantities of thick fibrils in a well-organized extracellular matrix (Fig. 2A, a). Treatment of MSC cultures with the osteogenic induction medium and resveratrol (various concentrations, 0.1, 1 and 10 µM) induced osteogenesis (Fig. 2A, b–d). However, no significant differences in osteogenesis were observed at the ultrastructural level between with resveratrol-treated and untreated MSC cultures.

In contrast, in the presence of the sirtuin inhibitor nicotinamide, osteogenesis was not observed, and some MSCs underwent apoptosis, with degeneration of the cells, membrane blebbing, nuclear damage and formation of apoptotic bodies. Remaining cells differentiated to adipocytes as demonstrated by lipid accumulation in fat vacuoles (Fig. 2A, e–g). The quantity of differentiated adipocytes in culture increased in the presence of 10 or 100 mM nicotinamide. Transmission electron microscopy clearly showed that the MSCs differentiated to adipocytes, accumulating cytoplasmic lipid droplets and exhibiting well-developed rough endoplasmic reticulum and mitochondria.

Pre-treatment of MSCs with resveratrol and co-treatment with nicotinamide promoted osteogenic differentiation (Fig. 2A, h–j). However, the inhibition of adipogenesis by resveratrol was concentration dependent. Pre-treatment of MSCs with 1 µM resveratrol and co-treatment with 100 mM nicotinamide resulted in adipogenesis.

Incubation of pre-osteoblastic MC3T3-E1 cells with the osteogenic induction medium or/and resveratrol resulted in osteogenesis. However, in contrast to MSCs, treatment of pre-osteoblastic MC3T3-E1 cells with nicotinamide, led to apoptosis instead of to formation of adipocytes. Pre-treatment of pre-osteoblastic MC3T3-E1 cells with resveratrol and co-treatment with nicotinamide promoted osteogenic differentiation (data not shown).

Statistical evaluation of the data clearly highlighted changes in the number of cells with fat vacuole accumulation before and after nicotinamide-treatment in MSC-osteogenesis high-density cultures. Co-treatment with resveratrol decreased the number of adipocytes with accumulated fat vacuoles (Fig. 2B).

### Effect of resveratrol or/and nicotinamide on extracellular matrix, Runx2 and PPAR-γ expression during MSC-osteogenesis and in pre-osteoblastic cell-osteogenesis

**Figure 3:**
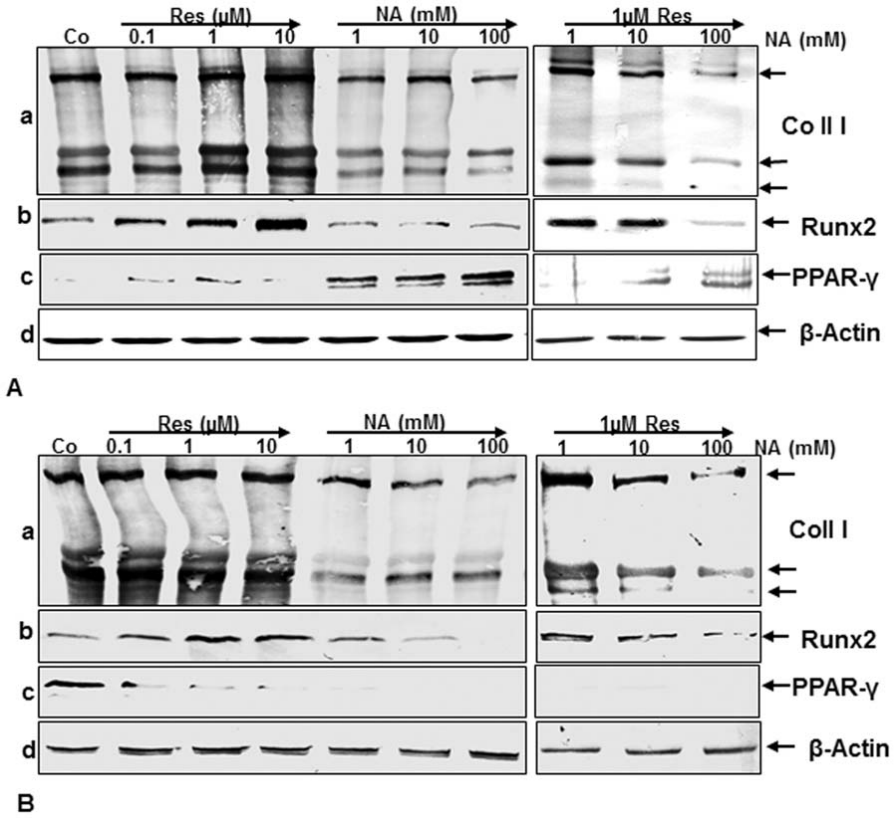
Effect of resveratrol or/and nicotinamide on extracellular matrix, Runx2 and PPAR-γ expression during osteogenesis of MSC and pre-osteoblastic cells in high-density cultures. Whole cell lysates (500 ng of protein per lane) were probed with antibodies for collagen type I (a), for the osteogenic specific transcription factor Runx2 (b) and for the adipogenic specific transcription factor PPAR-γ (c) in MSC (A) and in pre-osteoblastic cells in high-density culture (B). Cultures were treated with 0.1, 1 and 10 µM resveratrol alone, or with 1, 10 and 100 mM nicotinamide alone or pre-treated with 1 µM resveratrol for 4 hours and then co-treated with 1, 10, 100 mM nicotinamide or left untreated for 2 weeks with osteogenic induction medium in high-density cultures. Untreated cultures (without resveratrol or nicotinamide) produced collagen type I (a, A–B) and Runx2 (b, A–B) in both cultures. Incubation with nicotinamide reduced collagen type I and Runx2 production and increased the expression of PPAR-γ in a concentration dependent manner in MSC-cultures (c, A) and decreased the expression of PPAR-γ in a concentration dependent manner in pre-osteoblastic cultures (III, B). However, pre-treatment with resveratrol inhibited the adverse effects of nicotinamide and the osteoblasts produced large amounts of collagen type I and Runx2. Synthesis of the housekeeping protein β-actin was unaffected (d, A–B).

To confirm the morphological results described above and to demonstrate more precisely the identity of the osteogenesis or adipogenesis by MSCs or pre-osteoblastic cell cultures, whole cell extracts were probed for collagen type I, Runx2 and PPAR-γ. High collagen type I content was detected by immunoblotting in the osteogenic-induced control cultures. Treatment of MSCs with osteogenic induction medium and 0.1, 1 and 10 µM resveratrol in high-density cultures resulted in a stimulation of collagen type I production and expression of Runx2. MSC cultures treated with nicotinamide alone at various concentrations showed a significant downregulation of synthesis of collagen type I and Runx2, but up-regulation of PPAR-γ and this was in a concentration-dependent manner (Fig. 3A, a, b, c left panel). In contrast to this, pre-treatment of MSCs with resveratrol (1 µM, 4 h) followed by stimulation with the sirtuin inhibitor, nicotinamide (1, 10 and 100 mM) resulted in an inhibition of nicotinamide-induced effects on collagen type I production and Runx2 during MSC-osteogenesis and downregulated PPAR-γ in high-density cultures (Fig. 3A, a, b, c right panel). However, 1 µM resveratrol could not completely inhibit the blocking effect of 100 mM nicotinamide on the synthesis of collagen type I and Runx2 during osteogenesis and downregulated PPAR-γ in high-density culture (Fig. 3A, a, b, c right panel). Synthesis of the house-keeping protein β-actin remained unaffected (Fig. 3A, d).

To see that the nicotinamide-induced inhibition of Runx2 and stimulation of PPAR-γ and adipogenesis during MSC-osteogenesis occurs also transiently during osteogenesis with pre-osteoblastic cells, we compared the effects of resveratrol or/and nicotinamide on protein expression profiles of MSC and pre-osteoblastic MC3T3-E1 cells during the osteogenesis in high-density culture to further confirm their differentiation capacities. Pre-osteoblastic MC3T3-E1 cells produced large quantities of collagen type I in presence of 0.1, 1 and 10 µM resveratrol and Runx2 expression was also stimulated. High collagen type I content was also detected in the osteogenic-induced control cultures. Pre-osteoblastic cells treated with nicotinamide alone at various concentrations showed a significant downregulation of synthesis of collagen type I and Runx2. Interestingly, in opposite to MSC-cultures, when nicotinamide was added to pre-osteoblastic MC3T3-E1 cells, no significant effect was seen on formation of adipocytes and PPAR-γ expression compared with MSCs and this was in a concentration-dependent manner (Fig. 3B, a, b, c left panel). Moreover, pre-treatment of pre-osteoblastic MC3T3-E1 cells with resveratrol (1 µM, 4 h) followed by stimulation with nicotinamide (1, 10 and 100 mM) resulted in an inhibition of nicotinamide-induced effects on collagen type I production and Runx2 and downregulated PPAR-γ in high-density cultures (Fig. 3B, a, b, c right panel). However, 1 µM resveratrol could not completely inhibit the blocking effect of 100 mM nicotinamide on the synthesis of collagen type I and Runx2 in high-density culture (Fig. 3B, a, b, c right panel). Taken together, these results indicate that adipocytes and osteoblasts share a common progenitor, i.e. MSCs expressing PPAR-γ signaling can induce trans-differentiation of osteoblasts to adipocytes by inhibiting of Runx2, whereas, the pre-osteoblastic cells only have the capability to differentiate into osteoblasts.

### Expression of Sirt-1 in MSCs before and after osteoblastic differentiation *in vitro*

**Figure 4:**
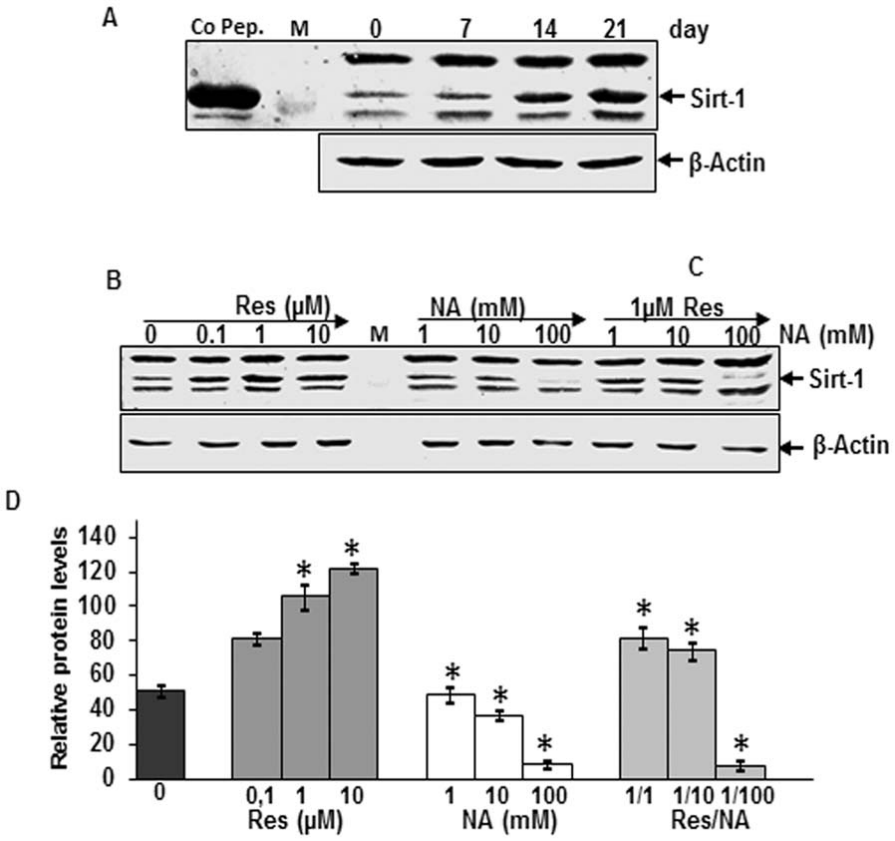
Effect of resveratrol on nicotinamide-induced inhibition of Sirt-1 expression. *A: Sirt-1 protein expression during osteogenesis in monolayer cultures.* 21 days monolayer cultures of osteogenic induced fat tissue derived MSCs. Whole cell lysates (500 ng/lane) were probed for Sirt-1. MSCs express high levels of Sirt-1 before and after induction of osteogenic differentiation. Synthesis of the housekeeping protein β-actin was unaffected. Sirt-1 control peptide was used as a control (co pep.). M = Marker for molecular weights. *B–C: Effect of resveratrol on NA-induced inhibition of Sirt-1 expression during osteogenesis in monolayer culture.* 14 days osteogenic induction culture of control MSCs, cells treated with 0.1, 1, 10 µM resveratrol or with 1, 10, 100 mM nicotinamide or pre-treated with 1 µM resveratrol for 4 h followed by co-treatment with nicotinamide. Whole cell lysates (500 ng/lane) were fractionated and subjected to western blotting with antibodies against Sirt-1. D: *Densitometric evaluation was performed for Sirt-1 expression from Fig. B–C.* Each experiment was performed in triplicate and mean values and standard deviation are indicated. Values were compared to the control and statistically significant values with *p*<0.05 were designated by an asterisk (*).

The NAD-dependent protein deacetylase Sirt-1 has been shown to attenuate development of adipocytes from pre-adipocytes through inhibition of PPAR-γ activity [39]. Next, we wanted to evaluate whether phytochemicals known to regulate the activity of Sirt-1 could influence the formation of MSCs during osteoblast differentiation *in vitro*. First, we could demonstrate the expression of Sirt-1 in the MSCs derived from fat tissue. Whole cell lysate from MSCs in monolayer cultures treated with osteogenic induction medium for 0, 7, 14 and 21 days were analyzed by western blot with anti-Sirt-1 antibody. Sirt-1 was expressed in the MSCs before (day 0) and after induction of osteoblastic differentiation (Fig. 4A).

### Resveratrol inhibits nicotinamide-induced down-regulation of Sirt-1 during osteogenic differentiation of MSCs *in vitro*

To investigate the possible mechanism for dedifferentiation of MSCs to adipocytes during osteogenesis, we investigated the effect of resveratrol on the expression of Sirt-1. As shown in Figures 4B–C, when cells were treated with sirtuin inhibitor, nicotinamide (1, 10, 100 mM), the expression of endogenous Sirt-1 was decreased in a concentration dependent manner. In contrast, resveratrol treatment caused an increase of the Sirt-1 expression in a concentration dependent manner (Fig. 4B). Interestingly, pre-treatment of MSCs with resveratrol (1 µM, 4 h) followed by stimulation with nicotinamide (1, 10 and 100 mM) caused a concentration dependent up-regulation in Sirt-1 expression (Fig. 4C). Densitometric analysis of a representative experiment performed in triplicate from the effect of resveratrol on nicotinamide-induced inhibition of Sirt-1 expression during osteogenesis in monolayer culture showed that the increasing concentration of nicotinamide decreased the amount of Sirt-1, whereas pre-treatment with resveratrol markedly increased it (Fig. 4D).

### Sirt-1 blocks adipogenesis by repressing PPAR-γ activity and NCoR involvement in this process

**Figure 5:**
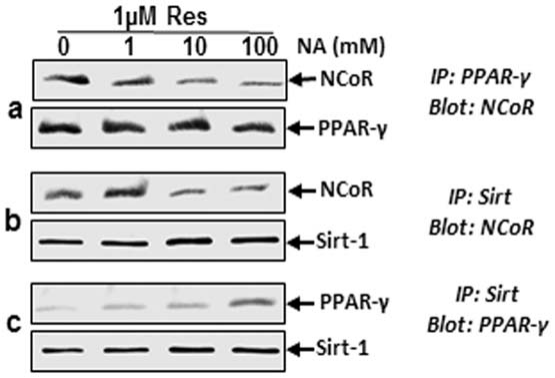
Effect of resveratrol and nicotinamide on association of Sirt-1 proteins with PPAR-γ and NCoR in MSC high-density cultures. Cultures were treated with 0, 1, 10, 100 mM nicotinamide or pre-treated with 1 µM resveratrol for 4 h followed by co-treatment with nicotinamide over 14 days with osteogenic induction medium. Cultures were lysed and immunoprecipitated with anti-PPAR-γ (a), or anti-Sirt-1 (b, c). The immunoprecipitates were separated by SDS-PAGE and analyzed by immunoblotting using anti-NCoR (a, b) and anti- PPAR-γ (c). The same blots were re-probed with an antibody to anti-PPAR-γ (a), anti-Sirt-1 (b, c). Results shown are representative of three independent experiments.

The nuclear receptor PPAR-γ is known to regulate adipogenesis [39]. It has also been shown that the nuclear receptor co-repressor, NCoR, binds to known PPAR-γ sites of promoters of adipogenic genes in differentiated 3T3-L1 adipocytes [39]. To test whether Sirt-1 is a PPAR-γ co-repressor by means of NCoR, we pre-treated the MSCs with resveratrol (1 µM, 4 h) followed by stimulation with nicotinamide (1, 10 and 100 mM) in high-density cultures, and co-immunoprecipitation assays. As shown in Figure 5a, after immunoprecipitation with anti-PPAR-γ antibodies, the samples were probed by immunoblotting with anti-NCoR. The results indicate that PPAR-γ interacts with NCoR and this interaction is dependent on the concentration of nicotinamide (Fig. 5a). As shown in Figure 5b–c, after immunoprecipitation with anti-Sirt-1 antibodies, the samples were probed by immunoblotting with anti-NCoR and anti-PPAR-γ. The results suggest that Sirt-1 interacts with NCoR and PPAR-γ. The expression of endogenous NCoR was decreased and the expression of endogenous PPAR-γ was increased in a concentration dependent manner with nicotinamide. Taken together, these results demonstrate that NCoR may, at least in part, be involved in repression of PPAR-γ by Sirt-1.

### Resveratrol blocks nicotinamide-induced inhibition of the association of Sirt-1 proteins with the early osteogenic transcription factor Runx2 in MSC high-density cultures

**Figure 6:**
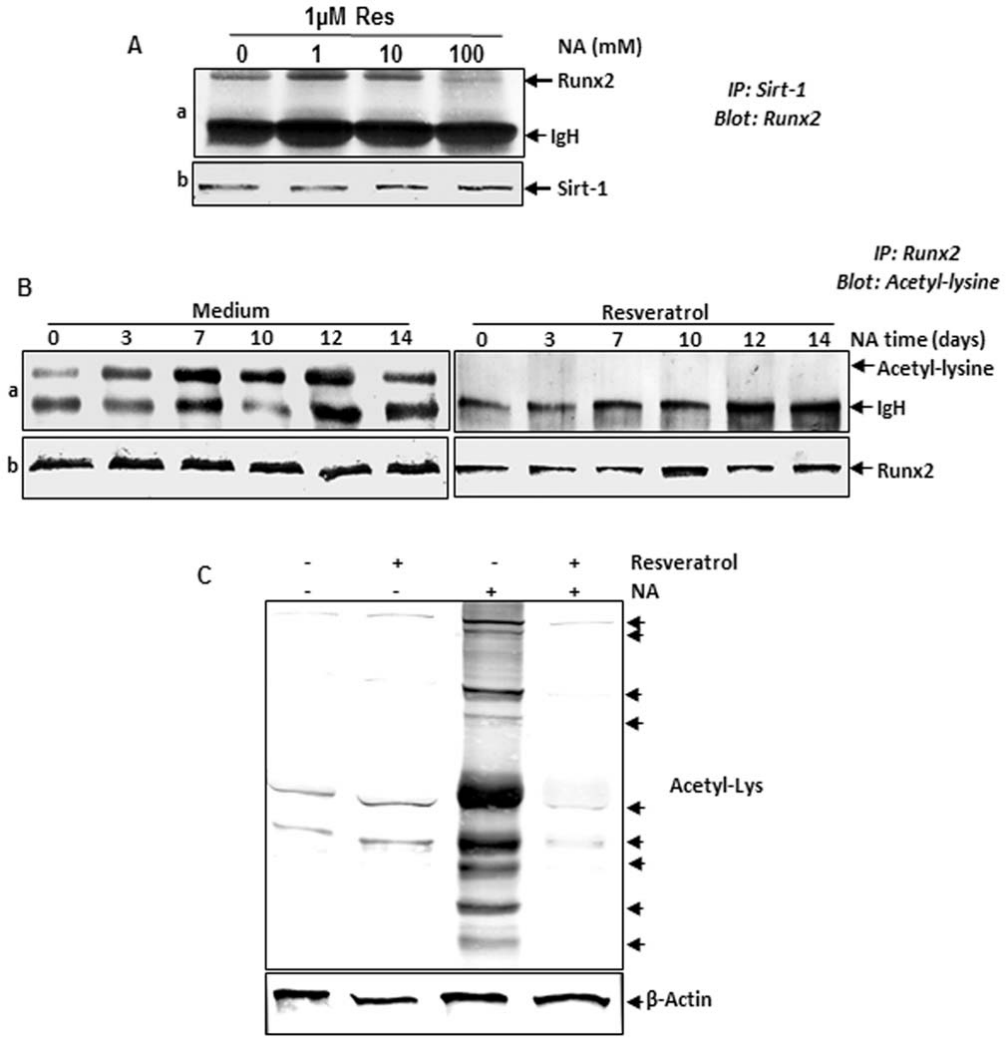
Association of Sirt-1 proteins with the early osteogenic transcription factor Runx2 during osteogenesis. *A: Effect of resveratrol and NA on association of Sirt-1 proteins with Runx2 during osteogenesis in MSC high-density cultures.* Cultures were pre-treated with 1 µM resveratrol for 4 h followed by co-treatment with nicotinamide 1, 10, 100 mM over 14 days with osteogenic induction medium. Cultures were lysed and immunoprecipitated with anti-Sirt-1 antibody. The immunoprecipitates were separated by SDS-PAGE and analyzed by immunoblotting using anti-Runx2 antibodies (a). The same blots were re-probed with an antibody to anti-Sirt-1 (b). Results shown are representative of three independent experiments. IgH, immunoglobulin heavy chain. *B: Effect of resveratrol on NA-induced acetylation of Runx2 during osteogenesis in MSC high-density cultures.* Cells were treated with 10 mM nicotinamide or pre-treated with 1 µM resveratrol for 4 h followed by co-treatment with 10 mM nicotinamide over 14 days with osteogenic induction medium. Whole-cell extracts were prepared, immunoprecipitated with an anti-Runx2 antibody, and subjected to western blot analysis using an anti–acetyl-lysine antibody (a). The same blots were re-probed with an antibody to anti-Runx2 (b). *C: Effect of resveratrol on NA-induced protein acetylation.* Cells were treated with 1 µM resveratrol for 4 h and then exposed to 10 mM nicotinamide over a period of 14 days with osteogenic induction medium. Whole cell extracts were prepared and subjected to western blot analysis using an anti-acetyl-lysine antibody. The same blots were re-probed with an antibody to β-actin.

To determine possible downstream signaling proteins during osteogenesis in high-density cultures, we examined whether Sirt-1 associates with the early osteogenic transcription factor Runx2 subsequently activating the pathway that stimulates osteogenesis. The fact that the expression pattern of Sirt-1 and Runx2 protein are similarly stimulated or inhibited by resveratrol or nicotinamide in MSCs (Fig. 4A, B; Fig. 3A, B), suggests that these two proteins could interact together. To examine this, we performed co-immunoprecipitation of endogenous Sirt-1 protein and precipitated Runx2. As shown in Figure 6A, the cells were pre-treated with resveratrol (1 µM, 4 h) and then co-treated with nicotinamide (1, 10 and 100 mM) for 14 days, then co-immunoprecipitation assays were performed. After immunoprecipitation with anti-Sirt-1 antibodies, the samples were probed by immunoblotting with anti-Runx2. The results indicated that Runx2 was co-immunoprecipitated by anti-Sirt-1 antiserum but not by pre-immune serum in high-density cultures (Fig. 6A). This interaction of Sirt-1 with Runx2 was decreased with as little as 10 mM nicotinamide and indicates that the expression and association of Runx2 with Sirt-1 is concentration-dependent. Taken together, these results indicate that during osteogenesis resveratrol activates Sirt-1 and induces Sirt-Runx2 complex formation, which activates the osteogenic pathway.

### Effect of resveratrol on nicotinamide-induced acetylation of Runx2 in MSC high-density cultures

Resveratrol has been shown to activate Sirt-1 deacetylase activity [18], [30]. The fact that the stimulation of Sirt-1 protein correlated with the expression of Runx2 and in addition, that both proteins interact together [30], suggests that Runx2 might be a substrate for Sirt-1 deacetylation. As shown in Fig. 6B, nicotinamide treatment strongly induced Runx2 lysine acetylation in a time dependent manner in high-density cultures. To examine the functional impact of Sirt-1 regulation of nicotinamide-mediated acetylation of Runx2, we pre-treated MSCs with resveratrol and then co-treated them with nicotinamide during osteogenesis in high-density cultures for the indicated time periods. Interestingly, the nicotinamide-induced acetylation of Runx2 markedly decreased by pre-treatment with resveratrol, suggesting, at least in part, a significant reduction in nicotinamide-induced Runx2 acetylation by Sirt-1 activity (Fig. 6B). To determine whether resveratrol is able to block the nicotinamide-induced acetylation of proteins, whole cell lysates from cells treated with nicotinamide, resveratrol or combination of both of them were analyzed by western blotting using anti-acetyl lysine antibody. As shown in Fig. 6C, nicotinamide induced acetylation of several proteins, whereas resveratrol suppressed the acetylation of these proteins. These findings suggest that resveratrol-activated Sirt-1 plays an important role in inhibiting nicotinamide-activated PPAR-γ/NCoR complex resulting in a decrease of Runx2 acetylation.

### Specific antisense oligonucleotides downregulate Sirt-1 in MSCs *in vitro*

**Figure 7:**
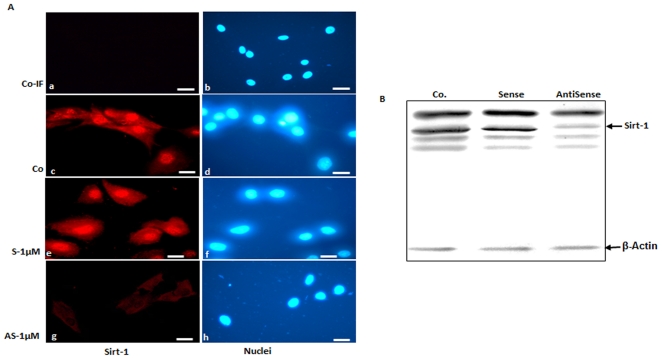
Specific antisense oligonucleotides against Sirt-1 in MSCs lead to decreased Sirt-1 expression in monolayer culture as revealed by immunofluorescence microscopy. A: Mesenchymal stem cells either served as controls (not treated, without primary antibody, a–b; not treated, with primary antibody, c–d) or were transfected with sense (e–f) or antisense (g–h) with 1 µM in the presence of lipofectin for 24 h and resistant colonies were collected. The collected MSCs were subjected to immunolabeling with Sirt-1 antibodies and rhodamine-coupled secondary antibodies. Counterstaining was performed with DAPI to visualize the cell nuclei. It shows that Sirt-1 antisense but not control sense knocks down Sirt-1 protein levels in the nuclei of cells as visualized using an epifluorescence microscope. Images shown are representative of three independent experiments. ×160. *B:* cell extracts generated from the cells in *A* were used for immunoblotting assay with Sirt-1 or β-actin (loading control) antibodies. The immunoblot shown is representative of three independent experiments.

To investigate whether specific antisense oligonucleotides against Sirt-1 inhibit Sirt-1 expression, MSCs were transfected with specific antisense or sense oligonucleotides derived from nucleotide sequence coding upstream part of catalytic domain of Sirt-1 protein. The immunofluorescence analysis (Fig. 7A) as well as the immunoblot assays (Fig. 7B) showed that the specific antisense oligonucleotides reduced the levels of Sirt-1 expression and nuclear localization. In contrast, the control sense oligonucleotide had no effect on Sirt-1 expression. The results indicated that treatment with Sirt-1 antisense oligonucleotides inhibited Sirt-1 expression specifically and concentration dependently (data not shown) and the inhibition was not related to non-specific gene-regulatory events.

### Downregulation of Sirt-1 expression by antisense oligonucleotides enhances Runx2 acetylation, PPAR-γ activation and inhibits expression of Runx2 target genes (osteocalcin) during osteogenic differentiation of MSCs in monolayer cultures

**Figure 8:**
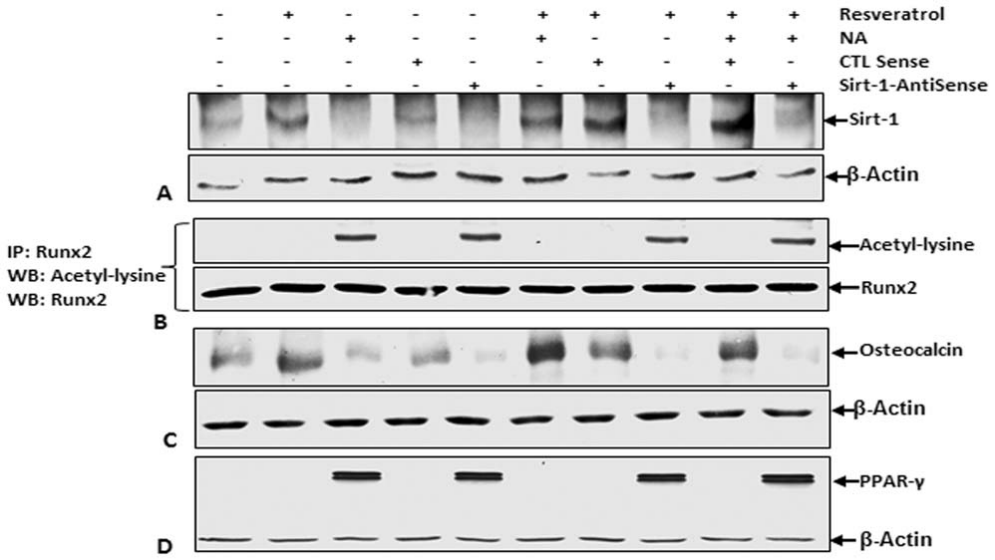
Effect of resveratrol on nicotinamide and/or antisense oligonucleotide-induced specific inhibition of Sirt-1 expression, Runx2 acetylation, Runx2 target genes (i.e.osteocalcin) or activation of PPAR-γ during osteoblastic differentiation of MSCs in monolayer culture. Cells were either untreated or treated with resveratrol (1 µM), nicotinamide (10 mM) or with Sirt-1 *antisense* (1 µM) or sense oligonucleotides (1 µM) in the presence of lipofectin alone or cells were pre-treated with resveratrol for 4 h followed by co-treatment with Sirt-1 antisense or sense oligonucleotides in the presence of lipofectin for 24 h or/and with nicotinamide over 21 days with osteogenic induction medium in monolayer cultures. (A) Whole cell lysates (500 ng/lane) were fractionated and subjected to western blotting with antibodies against Sirt-1 and β-actin. Synthesis of the housekeeping protein β-actin was unaffected. (B) Whole-cell extracts were prepared, immunoprecipitated with an anti-Runx2 antibody, and subjected to western blot analysis using an anti–acetyl-lysine antibody. The same blots were re-probed with an antibody to anti-Runx2. Whole cell lysates (500 ng/lane) were fractionated and analyzed by immunoblotting using anti-osteocalcin (C) or anti-PPAR-γ (D) antibodies and β-actin. Synthesis of the housekeeping protein β-actin was unaffected.

Based on the results of co-immunoprecipitation assays (Figure 6A), Sirt-1 interacts directly with Runx2 *in vitro*, which raises the possibility that Runx2 may be a substrate for Sirt-1 deacetylase. Since Sirt-1 acts as a protein deacetylase, next we examined whether the inhibitory effect of resveratrol on Runx2 acetylation is Sirt-1 dependent. The Sirt-1 specific oligonucleotide-transfected cells efficiently knocked down Sirt-1 protein levels during osteogenesis *in vitro* (Fig. 8A), and this abolished the ability of resveratrol (activator of Sirt-1) to deacetylate Runx2 in resveratrol and/or nicotinamide-stimulated cells (Fig. 8B) in monolayer cultures. Interestingly, the acetylation content of Runx2 was higher in cells treated with specific antisense oligonucleotides than in cells treated with or without sense oligonucleotides, suggesting the higher acetylated content of Runx2 protein is related to downregulated Sirt-1 expression and Runx2 could be a substrate for Sirt-1 deacetylase. To examine and establish a correlation between Sirt-1 and the activity of Runx2, western blot analysis with anti-osteocalcin (Runx2 target gene) antibody was performed. As shown in Fig. 8C, osteocalcin protein was downregulated in cells treated with nicotinamide and specific antisense oligonucleotides, compared to the cells treated with or without sense oligonucleotides. Taken together, these results suggest that downregulation of Sirt-1 in MSCs can decrease Runx2 activities and its downstream target genes.

To further investigate, if the acetylation of Runx2 by Sirt-1 downregulation, has an effect on the expression of PPAR-γ protein, western blot analysis with anti-PPAR-γ antibody was performed. As shown in Fig. 8C, PPAR-γ protein was elevated in cells treated with specific antisense oligonucleotides, compared to the cells treated with or without sense oligonucleotides. These results suggest that downregulation of Sirt-1 in MSCs can increase adipogenic differentiation and expression of the adipose transcription regulator PPAR-γ and modulate the expression of downstream target genes.

## Discussion

The aim of this study was to determine whether the naturally occurring phytoestrogen resveratrol can influence the osteoblastic differentiation of MSCs through its effects on Sirt-1-mediated cellular responses in an *in vitro* model of osteogenesis. This study leads to the following findings: (1) In the presence of nicotinamide (sirtuin inhibitor) some MSCs differentiated into adipocytes. (2) However, pre-treatment of MSCs with resveratrol protected them from the effects of nicotinamide-induced sirtuin inhibition resulting in osteogenesis (3) resveratrol blocked nicotinamide-induced inhibition of Runx2, a well-known important transcription factor involved in osteoblast recruitment and differentiation [40]. Further, resveratrol simultaneously inhibited the nicotinamide-induced fat transcription regulator PPAR-γ in the same cultures. (4) In opposite to MSCs, pre-osteoblastic cells treated with nicotinamide underwent apoptosis and did not differentiate to adipocytes, suggesting that adipocytes and osteoblasts share a common progenitor, while pre-osteoblastic cells only have the capability to differentiate into osteoblasts. (5) Resveratrol-activated Sirt-1 in MSCs increased its binding to PPAR-γ and repressed PPAR-γ activity. (6) The modulatory effects of resveratrol-activated Sirt-1 on nicotinamide-induced expression of PPAR-γ were found to be mediated, at least in part by the binding and deacetylation of Runx2. (7) Finally, we describe, for the first time, an antisense oligonucleotide approach to downregulate Sirt-1 expression in MSCs and demonstrate its ability to functionally inhibit osteogenesis and induce adipogenesis.

The differentiation capacity of MSCs is a highly investigated area of biology and medicine. However, little is known about the behavior of MSCs and progenitor cells during osteogenic differentiation. Therefore, we studied the differentiation of MSCs and pre-osteoblastic cells (MC3T3-E1) to compare their differentiation capacities. In the presence of resveratrol or/and nicotinamide, MSCs differentiate into osteoblasts and adipocytes in high-density cultures. In contrast to MSCs, pre-osteoblast cells were programmed to differentiate into their committed target osteoblast cells, as they were unable to differentiate into adipocytes [41]. For this reason, this study demonstrates that the primary isolated MSCs are stem cells, but pre-osteoblastic cells from the osteoblast progenitor MC3T3-E1 are not. In our study, MSCs treated with the sirtuin inhibitor (nicotinamide) downregulated bone-specific matrix compounds. Furthermore, the pre-treatment of MSCs with resveratrol lead to a recovery of osteoblastic differentiation and production of collagen type I in co-nicotinamide-stimulated MSCs. Thus, Sirt-1 appears to be a modulator of MSC differentiation to osteogenic cells. Moreover, in contrast to MSCs, pre-osteoblastic cells treated with nicotinamide downregulated bone-specific matrix components and cells underwent apoptosis.

Activation of Sirt-1 in MSCs decreases adipocyte differentiation and increases osteoblastic differentiation in high-density cultures. This differentiation was accompanied by expression of the osteoblastic transcription factor Runx2, which results in earlier initiation of the osteoblast differentiation programme. Since Sirt-1 inhibits the adipogenic transcription factor PPAR-γ, it also stimulates mechanisms regulating osteoblast differentiation. The most critical of these events is the activation of the master bone gene Runx2 [40]. Runx2 is responsible for expression of osteogenic marker genes, including osteopontin, osteocalcin and ALP. It has been reported that differentiation of MSCs to adipocytes can be inhibited by resveratrol and this process can be inhibited by the sirtuin blocker nicotinamide [18]. The mechanisms by which resveratrol and Sirt-1 mediate differentiation of MSCs to osteoblasts and inhibit adipogenesis, appear to involve, at least in part, the inhibition of PPAR-γ and activation of Runx2. Our co-immunoprecipitation data indicate that Sirt-1 interacts with the nuclear receptor PPAR-γ and this interaction was downregulated by nicotinamide. Moreover, we demonstrated that nuclear receptor PPAR-γ interacts with the nuclear receptor co-repressor NCoR. To test the possibility that Sirt-1 functionally represses PPAR-γ by the involvement of NCoR, we pre-treated the cells with resveratrol and co-treated with nicotinamide in high-density cultures. We found that PPAR-γ, NCoR and Sirt-1 were in a common complex, but in the presence of 1 µM resveratrol and 1 and 10 mM nicotinamide the amount of NCoR and Sirt-1 increased and the amount of PPAR-γ decreased. In contrast, in the presence of 1 µM resveratrol and 100 mM nicotinamide, the amount of Sirt-1 and NCoR decreased and the amount PPAR-γ increased in these experiments (Fig. 5). It has also been reported that Sirt-1 indirectly influences the transcriptional activity of the nuclear receptor PPAR-γ by docking the NCoR and SMRT to PPAR-γ [19]. The co-repressor protein, NCoR does not have an enzymatic activity, but it can activate the catalytic activity of histone deacetylases for deacetylation of histone proteins [42]. These data indicate that Sirt-1 interacts with the nuclear receptor co-repressor NCoR suggesting that Sirt-1, at least in part represses PPAR-γ activity by involving the co-activators. However, it should be considered that while resveratrol is known to activate Sirt-1, it has also other additional target proteins in the cells, thus it cannot be the only effect of Sirt-1.

Resveratrol's enhancement of osteogenesis was, at least in part regulated by Runx2 with additional contributions by Sirt-1. Resveratrol increases alkaline phosphatase activity in osteoblastic cells [10] an effect that is blocked by tamoxifen, an estrogen antagonist, suggesting that some of resveratrol's stimulatory actions may be mediated through the estrogen receptor. Gehm et al. have reported that resveratrol acts as a phytoestrogen (i.e. activating the estrogen receptor) and decreases osteoporosis [43]. Moreover, resveratrol is one of the most potent Sirt-1 activators; through binding to a special binding site it induces a conformational change in Sirt-1, lowering the K_m_ for both the acetylated substrate and NAD, thus resulting in increased enzymatic activity [18]. Sirt-1 facilitates the differentiation of MSCs to osteoblasts by directly regulating factors such as Runx2 and by modulation of nuclear receptor co-repressor NCoR and PPAR-γ.

It is known that the nuclear protein deacetylase Sirt-1 belongs to class III of histone deacetylases, resulting in transcriptional silencing. Thus, Sirt-1 participates in the regulation of genome architecture and gene expression [15], [16]. These results suggest that Runx2 and Sirt-1 directly interact together and that Runx2 might be a substrate for Sirt-1 deacetylation. Furthermore, our data demonstrate that nicotinamide treatment induced Runx2 acetylation and this was decreased and attenuated in the pre-treatment cultures with resveratrol, suggesting that Sirt-1 activity is increased in these cultures. This data suggest that resveratrol suppresses nicotinamide-induced Runx2 acetylation through Sirt-1 activation and at the same time through inhibition of NCoR/PPAR-γ complex.

Our study suggests that nicotinamide induces Runx2 acetylation in MSCs during osteogenesis *in vitro*. Runx2 acetylation was reversed by resveratrol, resulting in the suppression of nicotinamide-induced PPAR-γ transcriptional activity including adipogenesis. Resveratrol activates the deacetylase Sirt-1, but it can also inhibit a number of other signaling pathways [44], [45], [46], [47]. Therefore, we used a specific gene knockdown approach to investigate whether the ability of resveratrol to reverse Runx2 acetylation operates via Sirt-1. Knockdown of Sirt-1 protein levels inhibited the effects of resveratrol, suggesting that it was not operating via other signaling pathways. Furthermore, immunoprecipitation and western blotting demonstrated functional and physical interactions between Runx2 and Sirt-1, suggesting that Sirt-1 directly deacetylates Runx2. This is the first description of Runx2-Sirt-1 interactions; Sirt-1 mediated deacetylation of Runx2 suggests that this may play an important role in regulating resveratrol-activated Sirt-1 during osteogenesis. Additionally, the transcription factor Runx2 is modified by acetylation/deacetylation like other transcription factors such as p53, NF-kB, MyoD, HMG I, E2F and FOXO [30], [48], [49], [50].

**Figure 9:**
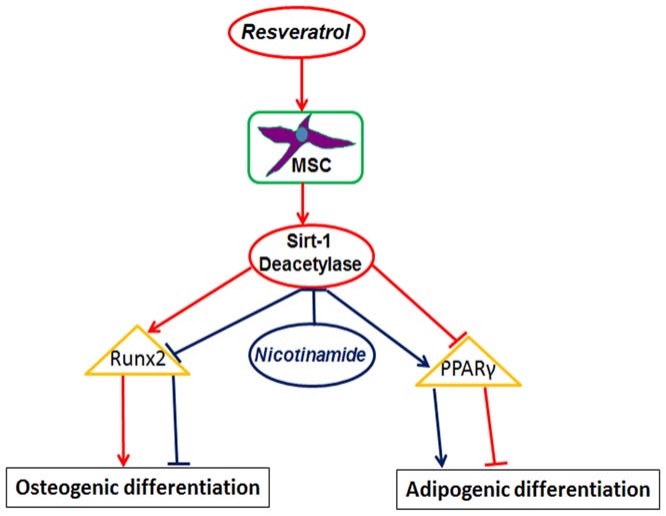
The cell signaling pathway for osteogenic/adipogenic differentiation in MSCs by resveratrol.

In summary, this study identified Runx2 acetylation as an important event in osteogenesis *in vitro*. Resveratrol-mediated inhibition of adipogenesis in MSCs was attributed to Sirt-1 activation, which deacetylated Runx2 and suppressed the nicotinamide-induced adipogenesis (Fig. 9). Thus, prevention or reversal of Runx2 acetylation may represent a new therapeutic strategy for suppression of osteoporosis.
